# The pivotal role of EMT-related noncoding RNAs regulatory axes in hepatocellular carcinoma

**DOI:** 10.3389/fphar.2023.1270425

**Published:** 2023-09-11

**Authors:** Alina-Veronica Ghionescu, Andrei Sorop, Simona Olimpia Dima

**Affiliations:** ^1^ Center of Excellence in Translational Medicine, Fundeni Clinical Institute, Bucharest, Romania; ^2^ Digestive Diseases and Liver Transplantation Center, Fundeni Clinical Institute, Bucharest, Romania; ^3^ Faculty of Medicine, Carol Davila University of Medicine and Pharmacy, Bucharest, Romania

**Keywords:** hepatocellular carcinoma, noncoding RNA, epithelial-mesenchymal transition, chemoresistance, exosomes

## Abstract

Hepatocellular carcinoma (HCC) remains a major health problem worldwide, being the leading cause of cancer-related deaths, with limited treatment options, especially in its advanced stages. Tumor resistance is closely associated with the activation of the EMT phenomenon and its reversal, being modulated by different molecules, including noncoding RNAs (ncRNAs). Noncoding RNAs have the potential to function as both tumor suppressors and oncogenic molecules, controlling the malignant potential of HCC cells. Basically, these molecules circulate in the tumor microenvironment, encapsulated in exosomes. Their impact on cell biology is more significant than originally expected, which makes related research rather complex. The temporal and spatial expression patterns, precise roles and mechanisms of specific ncRNAs encapsulated in exosomes remain primarily unknown in different stages of the disease. This review aims to highlight the recent advances in ncRNAs related to EMT and classifies the described mechanism as direct and indirect, for a better summarization. Moreover, we provide an overview of current research on the role of ncRNAs in several drug resistance-related pathways, including the emergence of resistance to sorafenib, doxorubicin, cisplatin and paclitaxel therapy. Nevertheless, we comprehensively discuss the underlying regulatory mechanisms of exosomal ncRNAs in EMT-HCC via intercellular communication pathways.

## 1 Introduction

Hepatocellular carcinoma (HCC) is a common lethal malignancy among patients with chronic liver disease, with approximately 800,000 deaths annually, according to the GLOBOCAN 2020 report (Sung et al., 2021). Several treatment options are available for therapeutic purposes, such as trans-arterial chemoembolization (TACE) with anthracyclines, cisplatin, and multikinase inhibitor, sorafenib (Pratama et al., 2019). However, these treatments become challenging to manage, due to the appearance of invasion, metastasis and recurrence, whose key molecular sign is EMT (Yan et al., 2018).

EMT (epithelial-mesenchymal transition) is a morphogenetic process in which epithelial cells get a mesenchymal phenotype. In early EMT, transcriptional factors (TFs) are activated to repress epithelial genes and activate the mesenchymal ones. These transcriptional changes trigger the following key events: cell-cell junction dissociations, apical-basal polarity loss, cytoskeleton architecture reorganization, the production of extracellular matrix (ECM) degradation enzymes, and cellular shape transformation. The activation of cellular pathways associates this process with proliferation, invasion, metastasis, and chemotherapy resistance (Yan et al., 2018; Dudas et al., 2020; Yang et al., 2020; Huang et al., 2022). Among these transformations, EMT is associated with numerous signaling pathways involved in inflammation, oncogenic and metabolic stress, hypoxia or apoptosis (Huang et al., 2022).

Moreover, many studies suggest that noncoding RNAs (ncRNAs), such as microRNAs (miRNAs), long-noncoding RNAs (lncRNAs) and circular RNAs (circRNAs), have been linked to both the EMT process activation and inhibition. Indeed, these types of RNAs have multiple roles in cancerous cells because one ncRNA transcript could target many molecules involved in different signaling pathways (Toden et al., 2021; Khanbabaei et al., 2022).

**FIGURE 1:**
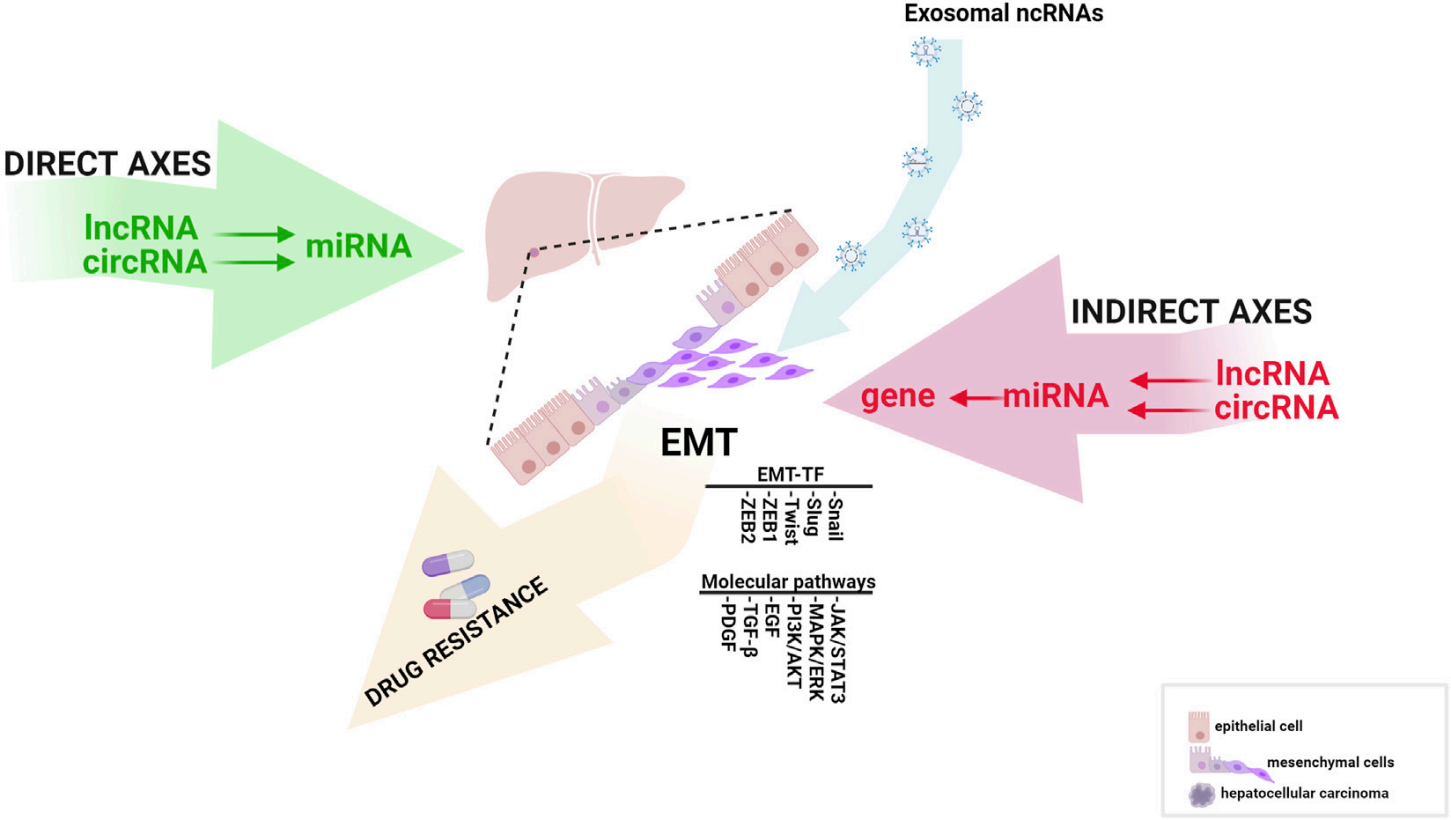
The influence of direct and indirect EMT-related ncRNA axes in HCC progression (created with biorender.com accessed on July 2023).

This review highlights ncRNAs’ significant direct and indirect signaling pathways in the EMT process and how these mechanisms are involved in HCC progression and chemoresistance. Finally, we provide an update on developing exosome-based therapies against HCC and their molecular aspects in EMT (Figure 1).

## 2 EMT-related ncRNAs mechanisms of action

As mentioned above, noncoding RNAs (ncRNAs), including microRNAs, lncRNAs and circRNAs, have oncogenic and tumor suppressor roles and regulate essential processes involved in cancer progression.

MicroRNAs (miRNAs) are noncoding single-stranded RNAs of approximately 22 nucleotides transcribed in pri-miRNA by RNA Pol II (Bartel, 2004). As described in the canonical pathway, Ribonuclease III and double-stranded-RNA-binding protein, DGCR8, recognize this structure in the nucleus, generating a pre-miRNA of ∼65 nucleotides. Pre-miRNA is exported to the cytoplasm by an Exportin 5 and Ran-GTP complex and recognized by RNase III Dicer, which forms a miRNA duplex. This mature form is incorporated into an RNA-induced silencing complex (RISC), directing RISC to complementary mRNA targets (Cai et al., 2004). In brief, miRNAs function as negative regulators of genes when binding to RNA 3′-untranslated region (3′-UTR) (Ha and Kim, 2014). Besides that, the interaction with coding sequences, gene promoters, and 5′-UTR has been proved (O'Brien et al., 2018). Because each miRNA can regulate multiple targets containing specific miRNA response elements (MREs) (Bassett et al., 2014) and play a crucial role in a variety of molecular processes, they have been studied in all cancer types (Esquela-Kerscher and Slack, 2006; Volinia et al., 2006; Nicoloso et al., 2009). In HCC, miRNAs modulate cell cycle, proliferation, apoptosis, epithelial-mesenchymal transition and metastasis (Sidhu et al., 2015). Furthermore, our previous studies have shown that miRNAs are an important tool in the prognostic and diagnostic HCC (Mjelle et al., 2019; Sorop et al., 2020).

Long noncoding RNAs (lncRNAs) are transcripts of approximately 200 nucleotides, which usually RNA Pol II transcribes, but so do RNA Pol I and RNA Pol III (Statello et al., 2021; Mattick et al., 2023). Moreover, they have a wide diversity, with an average of 100,000 human lncRNAs (Mattick et al., 2023). At first, lncRNAs were defined as transcriptional “junk” or “noise.” Still, in the past few years, more studies have shown the involvement of lncRNAs in different molecular pathways (Sun et al., 2017), indicating their interaction with DNA, RNA, or protein. The interaction mechanism could be: scaffold, decoy, guide, signal, or SINEUPs. Scaffold lncRNAs could act as archetype RNAs and are involved in the assembly of transcriptional regulators. The decoy mechanism implies acting as a competing endogenous RNA (ceRNA) or sponge of miRNAs, transcriptional factors, or RNA-binding proteins. In contrast, the guide mechanism involves the formation of a ribonucleoprotein complex, which targets a promoter or genomic loci (Rinn and Chang, 2012). Furthermore, lncRNAs could act as regulatory molecules (Nadhan et al., 2022) or SINEUPs containing SINE elements which enhance mRNAs translation (Toki et al., 2020).

Circular RNAs (circRNAs) are single-stranded RNAs with closed-loop structures and resistance to RNase R and exonucleases. They are generated from precursor RNA (pre-RNA) through back-splicing (Chen, 2016). This mechanism involves connecting a downstream donor site of a flanking downstream intron to an upstream acceptor site (Kristensen et al., 2019). Increasing research has revealed that circRNAs can sponge miRNAs, interact with proteins, interfere with transcription or splicing, or encode peptides (Zhang and Wang, 2021).

EMT plays a pivotal role in the early stage of metastasis (Bakir et al., 2020); thus, many studies have been conducted to determine the function of ncRNAs in this highly dynamic phenomenon. Therefore, this review underlines two types of mechanisms: direct and indirect.

### 2.1 Direct EMT-related ncRNAs’ mechanism of action

Direct mechanism involves direct interaction between miRNA and EMT-regulatory factors, such as twist family bHLH transcription factor 1 (TWIST), snail family transcriptional repressor 1 (SNAIL), or zinc finger E-box binding homeobox 1/2 (ZEB1/2) (Skovierova et al., 2018). We defined this mechanism by three crucial axes: miRNA/EMT, lncRNA/miRNA/EMT, and circRNA/miRNA/EMT.

Several miRNAs, such as miR-509-3p (Zhang et al., 2021), miR-361-5p (Yin et al., 2020), and miR-370-3p (Peng et al., 2022), have been found to inhibit TWIST1 expression via targeting its 3′UTR and to abate the EMT process. Li et al. (2022) observe that LINC00992 downregulates miR-361-5p and upregulates TWIST1, thus promoting cell proliferation, migration, and invasion. In addition, miR-370-3p decreases TWIST1 and SNAIL, affecting interleukin 8 (IL-8) expression and restraining the metastasis capacity in HCC cells (Peng et al., 2022). In contrast, LINC01133 (Yin et al., 2021) and lnc-UCID (Yuan et al., 2021) increase EMT by acting as a sponge of miRNAs, increasing SNAIL expression. Furthermore, circHIPK3 promotes metastases and ZEB2 expression via inhibiting miR-338-3p (Li et al., 2021). In contrast, circPTK2 and E-cadherin compete for binding miR-92a that, aggravates proliferation and invasion, while circPTK2 suppresses miRNA’s effect in HCC cells (Gong et al., 2020), as summarized in the direct mechanism part from Table 1.

**TABLE 1:** Summary of ncRNAs direct signaling pathways and their action on HCC tumor cell processes.

ncRNA	Expression	Target	Axis pathway	ncRNA involvement in cellular process	References
miR-509-3p	↓	TWIST	miR-509-3p/TWIST/EMT	(−) EMT, (−) proliferation, (−) metastasis	Zhang et al. (2021)
miR-361-5p	↓	TWIST1	miR-361-5p/TWIST1/EMT	(−) EMT, (−) proliferation, (−) migration, (−) invasion	Yin et al. (2020)
miR-370-3p	↓	TWIST1, SNAIL	IL-8/STAT3/miR-370-3p/TWIST1, SNAIL/EMT	(−) EMT, (−) metastasis	Peng et al. (2022)
LINC00992	↑	miR-361-5p	LINC00992/miR-361-5p/TWIST1	(+) EMT, (+) proliferation, (+) metastasis, (+) invasiveness	Li et al. (2022)
LINC01133	↑	miR-199a-5p	LINC01133/miR-199a-5p/SNAIL; LINC01133/ANXA2/STAT3/cyclin D1	(+) EMT, (+) proliferation, (+) migration, (+) invasion	Yin et al. (2021)
UCID	↑	miR-122, miR-203, miR-30b, miR-34a, miR-153	lnc-UCID/miR/SNAI1	(+) EMT, (+) metastasis, (+) migration, (+) invasion	Yuan et al. (2021)
circHIPK3	↑	miR-338-3p	circHIPK3/miR-338-3p/ZEB2	(+) EMT, (+) migration, (+) invasion, (+) metastases	Li et al. (2021)
circPTK2	↓	miR-92a	circPTK2/miR-92a/E-cadherin	(−) EMT, (−) proliferation, (−) invasion	Gong et al. (2020)

Note: downregulated expression (↓), upregulated expression (↑), inhibition of cellular process (−), enhance of cellular process (+).

### 2.2 Indirect EMT-related ncRNAs’ mechanism of action

The indirect mechanism involves miRNA/mRNA, lncRNA/miR/mRNA, and circRNA/miRNA/mRNA regulatory axes that modulate an EMT molecule.

#### 2.2.1 miRNA/mRNA axes

Numerous miRNA/mRNA axes have been found to be involved in the EMT process (Table 2).

**TABLE 2:** Summary of miRNAs signaling pathways and their action on HCC tumor cell processes.

miRNA	Expression	Target	Axis pathway	miRNA involvement in cellular process	References
miR-10a-5p	↓	SKA1	miR-10a-5p/SKA1	(−) EMT, (−) migration, (−) invasion, (−) tumor formation *in vivo*	Shen et al. (2021)
miR-143-3p	↓	FGF1	miR-143-3p/FGF1/EMT	(−) EMT, (−) proliferation, (−) invasion	Peng et al. (2021)
miR-139-5p	↓	WTAP	miR-139-5p/WTAP/EMT	(−) EMT, (−) invasion, (−) proliferation	Liu et al. (2021)
miR-181 a/b/c/d	↑	CDX2, GATA6, NLK1	miR-181/CDX2, GATA6, NLK1	(+) stemness	Ji et al. (2009)
miR-181b	↑	TIMP3	miR-181b/TIMP3/TGF- β	(+) migration, (+) invasion, (+) tumor formation *ex vivo*	Wang et al. (2010)
miR-181a	↑	BIM	mir-181a/TGF- β/EMT	(+) EMT	Brockhausen et al. (2015)
miR-181ab1	↑	CBX7	mir-181/TGF- β/EMT	(+) EMT, (+) proliferation	Chen et al. (2022)
miR-23b-3p	↓	c-MET	miR-23b-3p/c-MET/TGF-β1/EMT	(−) EMT, (−) migration, (−) invasion	Park et al. (2022)
miR-4521	↓	FAM129A	miR-4521/FAM129A/EMT	(−) EMT, (−) migration, (−) proliferation, (+) apoptosis	Ayesha et al. (2022)
miR-7	↓	BCL2L1	miR-7/BCL2L1/P53/EMT	(−) EMT, (−) proliferation, (−) metastasis	Zhang et al. (2023)
miR-22-3p	↓	SPRY2	miR-22-3p/CBL/SPRY2/ERK/EMT	(−) EMT, (−) migration, (−) invasion, (−) Cancer stem cell features	Zeng et al. (2020); Cui et al. (2023)
miR-383	↓	RBM3	miR-383/RBM3/STAT3/EMT	(−) EMT	Zhang et al. (2022)

Note: downregulated expression (↓), upregulated expression (↑), inhibition of cellular process (−), enhance of cellular process (+).

For instance, Shen et al. (2021) have found that miRNA-10a-5p is downregulated in HCC tissues and decreases EMT in HCC cells by targeting spindle and kinetochore-associated complex subunit 1 (SKA1). SKA1 is upregulated in tumors, promoting cancer progression, and has a prognostic value in HCC (Chen et al., 2018; Song et al., 2022). Other oncosuppressors are miR-143-3p and miR-139-5p, which repress fibroblast growth factor 1 (FGF1) and Wilms’ tumor 1-associating protein (WTAP). Those proteins increase EMT, proliferation and invasion of HCC cells (Liu et al., 2021; Peng et al., 2021). Moreover, Zhu et al. (2021) declare that miR-139-5p is regulated by lncRNA TTN antisense RNA 1 (TTN-AS1) and inhibits Sparc/osteonectin, cwcv, and kazal-like domains proteoglycan 1 (SPOCK1), an oncogenic proteoglycan involved in EMT (Vancza et al., 2022).

Growing studies have supported the importance of transforming growth factor beta (TGF-β) in HCC via SMAD/non-SMAD-dependent signaling pathways, which induce EMT-TFs (Hao et al., 2019). Several studies have shown that the miR-181 family positively correlates with TGF-β pathways, thus increasing EMT, tumor progression and stemness (Ji et al., 2009; Wang et al., 2010; Brockhausen et al., 2015; Chen et al., 2022). In contrast, miR-23b-3p has been proven to inhibit TGF- β1-induced EMT and block invasion and migration (Park et al., 2022).

Apoptosis or programmed cell death is a complex mechanism that involves death receptors (extrinsec pathway) and mitochondria (intrinsic pathway), by which it maintains cell homeostasis (Schattenberg et al., 2011). As discussed above, EMT confers resistance to apoptosis (Valdes et al., 2002). Interestingly, miR-4521 acts as an oncosuppressor in HCC cells by modulating mechanisms involved in proliferation and apoptosis. On the one hand, miR-4521 activates two apoptosis pathways (p-FAK/p-Akt/MDM2/P53 and FAK/p-Akt/BCL-2/BAX/Cytochrome-C/Caspase-3/Caspase-9) by decreasing the expression of family with sequence similarity 129 member A (FAM129A); on the other hand, it thereby attenuates invasivity by blocking TIMP-1/MMP9/MMP2, p-FAK/p-Akt and EMT pathways (Ayesha et al., 2022).

Moreover, the miR-7/BCL2L1/P53 and miR-22-3p/CBL/SPRY2/ERK axes decrease EMT, invasion, proliferation and migration (Cui et al., 2023; Zhang et al., 2023). Another EMT inhibitor is miR-383, which negatively regulates the multi-functional RNA-binding protein (RBM3) expression. As reported, RBM3 upregulates signal transducer and activator of transcription 3 (STAT3) expression via binding to its mRNA (Zhang et al., 2022). In addition, STAT3 targets the TWIST promoter and positively regulates its transcriptional activity in HCC cells, thus inducing EMT (Zhang et al., 2015).

Moreover, many studies highlight indirect mechanisms that imply lncRNA/miRNA/mRNA and circRNA/miRNA/mRNA axes.

#### 2.2.2 lncRNA/miRNA/mRNA axes

Table 3 shows the lncRNA/miRNA/mRNA axes related to EMT in HCC. According to their oncological role, lncRNAs could be classified into two groups: onco-suppressor and oncotargets. Therefore, within the last 3 years, five lncRNAs, TMEM220-AS1 (Cao et al., 2021), lncRNA miR503HG (Song and Qiu, 2021), LINC02362 (Li et al., 2022), LINC02027 (Wang et al., 2023) and SATB2-AS1 (Huang et al., 2023), have been documented to function as miRNA sponge, to decrease a gene that promotes the EMT process. For instance, Huang et al. (2023) show that SATB2-AS1 is observably reduced in HCC tissues compared to adjacent tissues and its overexpression hampers tumor growth and metastasis *in vitro*. Besides, SATB2-AS1 also acts as a ceRNA for miR-3678-3p. This miRNA accelerates cell proliferation and suppresses cell apoptosis by blocking GRIM-19 (gene associated with retinoic-interferon-induced mortality 19), a negative STAT3/HIF-1α pathway regulator (Huang et al., 2023).

**TABLE 3:** Summary of lncRNAs signaling pathways and their influence in HCC tumor cells processes.

lncRNA	Expression	Target	Axis pathway	lncRNA involvement in cellular process	References
TMEM220-AS1	↓	miR-484	lnc-TMEM220-AS1/miR-484/MAGI1	(−) EMT, (−) proliferation, (−) invasion, (−) metastasis, (−) tumor growth, (+) apoptosis	Cao et al. (2021)
miR503HG	↓	miR-15b	lncRNA miR503HG/miR-15b/PDCD4	(−) EMT, (−) angiogenesis, (−) migration, (−) invasion	Song and Qiu (2021)
LINC02362	↓	miR-516b-5p	LINC02362/miR-516b-5p/SOCS2	(−) EMT, (−) proliferation, (−) migration, (−) invasion, (+) apoptosis	Li et al. (2022)
LINC02027	↓	miR-625-3p	LINC02027/miR-625-3p/PDLIM5	(−) EMT, (−) proliferation, (−) migration, (−) invasion	Wang et al. (2023)
SATB2-AS1	↓	miR-3678-3p	lnc-SATB2-AS/miR-3678-3p/GRIM-19/STAT3/HIF-1α	(−) EMT, (−) proliferation, (−) invasion, (−) migration, (−) metastasis, (−) tumor growth, (+) apoptosis	Huang et al. (2023)
LINC00668	↑	miR-532-5p	LINC00668/miR-532-5p/YY1	(+) EMT, (+) proliferation, (+) migration, (+) invasion	Xuan et al. (2020)
LINC00922	↑	miR-424-5p	LINC00922/miR-424-5p/ARK5	(+) EMT, (+) proliferation, (+) migration, (+) invasion	Ye et al. (2021)
UNC5B-AS1	↑	miR-4306	UNC5B-AS1/miR-4306/KDM2A	(+) EMT, (+) proliferation, (+) migration	Huang et al. (2021)
BACE1-AS	↑	miR-377-3p	lnc-BACE1-AS/miR-377-3p/CELF1	(+) EMT, (+) invasion, (+) migration, (+) metastasis	Liu et al. (2021)
DUXAP8	↑	miR-9-3p	lnc-DUXAP8/miR-9-3p/IGF1R	(+) EMT, (+) proliferation, (+) migration, (+) invasion	Guan et al. (2021)
LOC554202	↑	miR-485-5p	LOC554202/miR-485-5p/BSG	(+) EMT, (+) proliferation, (+) migration, (+) invasion	Yang et al. (2022)
SNHG1	↑	miRNA-376a	lnc-SNHG1/miR-376a/FOXK1/SNAIL	(+) EMT, (+) proliferation, (+) migration, (+) invasion, (−) apoptosis	Meng et al. (2021)
HAGLROS	↑	miR-26b-5p	lnc-HAGLROS/miR-26b-5p/KPNA2/p53	(+) EMT, (+) proliferation, (+) migration, (+) invasion, (−) apoptosis	Tang et al. (2022)
DARS-AS1	↑	miR-3200-5p	lnc-DARS-AS1/miR- 3200-5p/CKAP2/FAK/ERK	(+) EMT, (+) proliferation, (+) migration, (+) invasion, (+) cell growth, (+) metastasis, (−) apoptosis	Feng et al. (2021)
SNHG12	↑	miR-516a-5p	lnc-SNHG12/miR-516a-5p/HEG1	(+) EMT, (+) proliferation, (+) migration, (+) invasion, (−) apoptosis	Chen et al. (2021)
PRR34-AS1	↑	miR-296-5p	lnc-PRR34-AS1/miR-296-5p/E2F2/SOX12/Wnt/beta-catenin	(+) EMT, (+) proliferation, (+) migration, (+) invasion, (+) tumor growth	Qin et al. (2021)
NUTM2A-AS1	↑	miR-186-5p	lnc-NUTM2A-AS1/mIR-186-5p/KLF7/Wnt/beta-catenin	(+) EMT, (+) invasion, (+) cell growth, (+) stemness, (−) apoptosis	Long et al. (2023)
LINC01278	↑	miR-1258	β-catenin/TCF-4/LINC01278/miR-1258/SMAD2/3	(+) EMT, (+) invasion, (+) migration, (+) metastasis	Huang et al. (2020)
CRNDE	↑	miR-539-5p	lnc-CRNDE/miR-539-5p/POU2F1/AKT/NF-kB	(+) EMT, (+) proliferation, (+) migration, (+) invasion	Li et al. (2020)
HCP5	↑	miR-29b-3p	lnc-HCP5/miR-29b-3p/DNMT3A/AKT	(+) EMT, (+) invasion, (+) cell growth, (+) metastasis, (−) apoptosis	Zhou et al. (2021)
KDM4A-AS1	↑	miR-411-5p	lnc-KDM4A-AS1/miR-411-5p/KPNA2/AKT/HIF-1α	(+) EMT, (+) proliferation, (+) migration, (+) invasion, (+) metastasis, (+) tumor growth	Chen et al. (2021)
MAPKAPK5-AS1	↑	miR-154-5p	lnc-MAPKAPK5-AS1/miR-154-5p/PLAGL2/EGRT/AKT/HIF-1α	(−) EMT, (−) proliferation, (+) apoptosis, (−) metastasis	Wang et al. (2021)
TTN-AS1	↑	miR-139-5p	lnc-TTN-AS1/miR-139-5p/SPOCK1	(+) EMT, (+) proliferation, (+) migration, (+) invasion, (+) metastasis, (+) tumor growth, (−) apoptosis	Zhu et al. (2021)

Note: downregulated expression (↓), upregulated expression (↑), inhibition of cellular process (−), enhance of cellular process (+).

On the other hand, several lncRNAs increase EMT by sponging miRNAs that target oncogenes. LncRNAs such as LINC00668 (Xuan et al., 2020), LINC00922 (Ye et al., 2021), UNC5B-AS1 (Huang et al., 2021), BACE1-AS (Liu et al., 2021), DUXAP8 (Guan et al., 2021) and LOC554202 (Yang et al., 2022) were upregulated in HCC to contribute to specific lncRNA/miR/mRNA axes induced EMT. SNHG1 is another lncRNA with high expression levels in HCC; it is negatively correlated to a poor patient prognosis. SNHG1 regulates cell proliferation and invasion via EMT through miR-376a binding to elevate forkhead box protein K1 (FOXK1) expression, a molecule that binds and upregulates SNAIL (Meng et al., 2021). HAGLROS knockdown impaired HCC tumorigenesis *in vitro* and *in vivo*. HALGROS increases the karyopherin α2 (KPNA2) level and suppresses p53 signaling to abate apoptosis by acting as a miR-26b-5p sponge (Tang et al., 2022). DARS-AS1 induces EMT via interacting with miR-3200-5p, further promoting Cytoskeleton associated protein 2 (CKAP2) expression and FAK/ERK pathway activation (Feng et al., 2021).

SNHG12 (Chen et al., 2021), PRR34-AS1 (Qin et al., 2021) and NUTM2A-AS1 (Long et al., 2023) axes induce EMT via Wnt/β-catenin signaling. Furthermore, Huang et al. (2020) point out that miR-1258 is downregulated in HCC patients. *In vivo,* experiments showed that the miR-1258 overexpression in nude mice impeded metastatic lung nodule formation. At the molecular level, LINC01278 acts as a sponge of miR-1258 and upregulates SMAD2/3, thus suppressing E-cadherin and enhancing vimentin expression. Moreover, transcription factor 4 (TCF-4) binds to the promoter site of LINC01278 and increases β-catenin expression, TGF-β and Wnt/β-catenin pathways, thereby activating the LINC01278/miR-1258/Samd2/Smad3 axis (Huang et al., 2020).

CRNDE and HCP5 induce Akt pathway activation by sponging miR-539-5p andmiR-29b-3p, respectively, to promote the EMT and the progression of HCC (Li et al., 2020; Zhou et al., 2021). Furthermore, two others oncogenic lncRNAs, KDM4A-AS1 and MAPKAPK5-AS1, activated by hypoxia-inducible factor 1-alpha (HIF1α), have also been found to increase protein kinase B (Akt) (Chen et al., 2021; Wang et al., 2021); their corresponding axes being listed in Table 3.

#### 2.2.3 circRNA/miRNA/mRNA axes

In the Table 4 there are highlighted critical pathways that involve circRNAs. In HCC, the levels of circFGGY (circ_0006633) (Feng et al., 2022), circ_0000098 (Li et al., 2021), and circEPB41L2 (Chen et al., 2021) are downregulated in tumor tissues and inhibit EMT, proliferation, migration, and invasion. In summary, authors highlight circFGGY/miR-545-3p/Smad7 (Feng et al., 2022), circ_0000098/miR-1204/ALX4 (Li et al., 2021) and circEPB41L2/miR-590-5p (Chen et al., 2021) axes as being important in HCC. Furthermore, Wu et al. (2020) revealed that circ_0004913 was downregulated in HCC tissues and that the overexpression of circ_0004913 constrained proliferation, EMT and metastasis by acting as a sponge of miR-184 and promoting hepcidin antimicrobial peptide (HAMP) expression. In brief, the circ_0004913/miR-184/HAMP axis regulates JAK2/STAT3/Akt signaling in HCC cells (Wu et al., 2020).

**TABLE 4:** Summary of circRNAs signaling pathways and their influence in HCC tumor cells processes.

circRNA	Expression	Target	Axis pathway	circRNA involvement in cellular process	References
circFGGY	↓	miR-545-3p	circFGGY/miR-545-3p/SMAD7	(−) EMT, (−) invasion, (−) migration, (−) cell growth	Feng et al. (2022)
circ_0000098	↓	miR-1204	circ_0000098/miR- 1204/ALX4	(−) EMT, (−) proliferation, (−) migration, (−) invasion	Li et al. (2021)
circEPB41L2	↓	miR-590-5p	circEPB41L2/miR-590-5p	(−) EMT, (−) proliferation, (−) migration, (−) invasion, (−) metastasis	Chen et al. (2021)
circ_0004913	↓	miR-184	circ_0004913/miR-184/HAMP	(−) EMT, (−) proliferation, (−) migration, (−) invasion, (−) tumor growth	Wu et al. (2020)
circ_0003998	↑	miR-143-3p	circ_0003998/miR-143 -3p/FOSL2; circ_0003998/miR-143 -3p/PCBP1/CD44v6	(+) EMT, (+) migration	Song et al. (2020)
circ_0101145	↑	miR-548c-3p	circ_0101145/miR-548c-3p/LAMC2	(+) EMT, (+) proliferation, (+) migration, (+) metastasis	Jin et al. (2020)
circBACH1	↑	miR-656-3p	circBACH1/miR-656-3p/SERB1	(+) EMT, (+) proliferation, (+) migration, (+) invasion, (+) tumor growth, (−) apoptosis	Li et al. (2021)
circPUM1	↑	miR-1208	circPUM1/miR-1208/MAP3K2	(+) EMT, (+) migration, (+) invasion	Zhang et al. (2021)
circ_0051040	↑	miR-569	circ_0051040/miR-569/ITGAV	(+) EMT, (+) proliferation, (+) migration, (+) invasion, (+) tumor growth, (+) metastasis	Ju et al. (2022)
circ_0001459	↑	miR-6165	circ_0001459/miR-6165/IGF1R	(+) EMT, (+) proliferation, (+) migration, (+) invasion, (+) tumor growth, (+) metastasis	Shen et al. (2022)
circSEC24A	↑	miR-421	circSEC24A/miR-421/MMP3	(+) EMT, (+) proliferation, (+) invasion, (+) migration, (+) cell growth	Zhang and Zhou (2022)
		miR-455-3p	circSEC24A/miR-455-3p/PPM1F	(+) EMT, (+) proliferation, (+) invasion, (+) metastasis, (+) tumor growth, (−) apoptosis	Liao et al. (2021)
circ_0003288	↑	miR-145	circ_0003288/miR-145/PD-L1	(+) EMT, (+) migration, (+) invasion	Xu et al. (2021)
circ_0091579	↑	miR-136- 5p	circ_0091579/miR-136-5p/TRIM27	(+) EMT, (+) proliferation, (+) migration, (+) invasion, (+) cell cycle progression	Mao et al. (2022)
circTOLLIP	↑	miR-516a-5p	circTOLLIP/miR-516a-5p/PBX3/EMT	(+) EMT, (+) proliferation, (+) metastasis	Liu et al. (2022)
circCDR1as	↑	miR-1287	circCDR1as/miR-1287/Raf1 and MEK/ERK	(+) EMT, (+) proliferation, (+) metastasis	Zhang et al. (2020)
circ-TLK1	↑	miR-138-5p	circTLK1/miR-138-5p	(+) EMT, (+) proliferation, (+) migration, (+) invasion	Lu et al. (2022)
circFoxo3	↑	miR-199a-5p	circFoxo3/miR-199a-5p/ABCC1	(+) EMT, (+) invasion, (+) tumor growth	Huang et al. (2020)

Note: downregulated expression (↓), upregulated expression (↑), inhibition of cellular process (−), enhance of cellular process (+).

In contrast, six circRNAs, circ_0003998 (Song et al., 2020), circ_0101145 (Jin et al., 2020), circBACH1 (Li et al., 2021), circPUM1 (Zhang et al., 2021), circ_0051040 (Ju et al., 2022) and circ_0001459 (Shen et al., 2022), have been observed to manipulate various miR/mRNA axes to induce EMT. Besides, elevated level of circSEC24A leads to the expression of protein phosphatase, Mg2+/Mn2+ dependent 1F (PPM1F) and matrix metalloproteinase 3 (MMP3) by sponging miR-455-3p and miR-421, respectively (Liao et al., 2021; Zhang and Zhou, 2022). MMPs are a class of enzymes that degrade extracellular matrix (ECM) proteins (Klein and Bischoff, 2011). In HCC, it was reported that MMP3 promotes EMT and metastasis (Scheau et al., 2019).

Circ_0003288 is an oncogenic RNA that enhances EMT by increasing programmed death-ligand 1 (PD-L1) and Akt pathways via miR-145 sponging (Xu et al., 2021). Circ_0091579 has been demonstrated to pin HCC patients and its downregulation inhibits EMT and promotes apoptosis *in vitro*. Also, miR-136-5p is a direct target of circ_0091579 and its overexpression suppresses the malignant potential of HCC cells via regulating tripartite motif containing 27 (TRIM27) expression (Mao et al., 2022).

Moreover, the Toll interacting protein (TOLLIP)-derived circRNA (circTOLLIP) is also found to be involved in the EMT of HCC. CircTOLLIP is upregulated in HCC via eukaryotic translation initiation factor 4A3 (EIF4A3), an RNA-binding protein. This circRNA acts as a ceRNA for miR-516a-5p, thus upregulating PBX3 and exhibiting pro-tumor roles *in vitro* and *in vivo* (Liu et al., 2022).

CircRNA CDR1as is highly expressed in some cancers (Jiang et al., 2020). Specifically, circRNA CDR1as is overexpressed in HCC tissues and its expression positively regulates EMT, proliferation and metastasis in HCC cells via the miR-1287 sponge. This circRNA enhances Raf-1 proto-oncogene, serine/threonine kinase (RAF1) expression, a crucial molecule in the RAS/RAF/MEK/ERK pathway (Zhang et al., 2020).

## 3 The role of ncRNA/mRNA axes in HCC drug resistance

As discussed above, EMT is associated with chemotherapy resistance by avoiding cell death mechanisms (De Las Rivas et al., 2021). Therefore, a growing number of studies have supported the importance of EMT-related ncRNAs in molecular pathways of different therapies (He et al., 2022).

Sorafenib is the first-line FDA-approved treatment for HCC (Niu et al., 2021) and an oral multikinase inhibitor that targets vascular endothelial growth factor receptor 2 (VEGFR2), platelet-derived growth factor receptor (PDGFR), hepatocyte factor receptor (KIT), or other molecules to decrease angiogenesis. HCC cells acquire resistance to sorafenib by different molecular pathways, including EMT (Marisi et al., 2018; Tang et al., 2020). In this context, lncH19 knockdown has been reported to inhibit EMT in HCC cells by enhancing miR-675 expression, which is involved in sorafenib sensitivity. In brief, H19 promoted sorafenib resistance (Xu et al., 2020). LncRNA-POIR also has an oncogenic effect and suppresses miR-182-5p expression, inhibiting the EMT process and triggering sorafenib sensitivity (Chen et al., 2021). Additionally, small nucleolar RNA host gene 3 (SNHG3) induces EMT and CD151 expression by functioning as a ceRNA for miR-128. LncRNA-SNHG3 can induce sorafenib resistance and promote invasion *in vitro* (Zhang et al., 2019). In contrast, lncLIMT (LINC01089), which reppresses miR-665 expression and EMT, decreases sorafenib resistance. In addition, LIMT inhibits tumor growth *in vivo* in tumor nude mouse models (Sun et al., 2022). MiR-125b-5p is upregulated in sorafenib-resistant HCC cell lines and its overexpression induces EMT by repressing ataxin 1 (ATXN1) expression. Thus, it was reported that miR-125b-5p enhances sorafenib resistance *in vivo* (Hirao et al., 2021).

Besides Sorafenib, TACE with doxorubicin and cisplatin is used in HCC advanced patients (Lu et al., 2017; Couri and Pillai, 2019).

Doxorubicin (Adriamycin, DOX) is an anthracycline drug used as an antineoplastic agent. The most known mechanism of action involves the interaction with topoisomerase IIα (TOP2A) (Tewey et al., 1984) and the activation of apoptosis (Roos and Kaina, 2013). Anthracycline drug resistance is caused by the incapability of DOX to accumulate in the nucleus (Cox and Weinman, 2016). For instance, Zhang et al. (2021) reported that overexpression of linc-ROR (long intergenic non-protein coding RNA (linc)-regulator of reprogramming) increases DOX resistance in HCC cell lines by TWIST upregulation. Also, circFoxo3 has higher expression in adriamycin-resistant patients. It has been shown that circFoxo3 via miR-199a enhances ABCC1 expression, a known protein involved in drug resistance. Moreover, the downregulation of miR-199a promoted EMT signaling in HCC cells and reversed circFoxo3 inhibition effects (Huang et al., 2020).

Li et al. (2020) identified that circ_0003998 downregulation facilitated DOX-sensitivity by E2F Transcription Factor 3 (E2F3) regulation. They further identified circ_0003998 as a sponge of miR-218-5p and Eukaryotic initiation factor 5A2 (EIF5A2) as a direct target of miR (Li et al., 2020). Moreover, EIF5A2 is involved in genistein resistance, an essential anti-tumoral phytoestrogen that promotes apoptosis (Sarkar and Li, 2002) and inhibits EMT and stemness. MiR-1275 is a tumor suppressor that can bind 3′-UTR EIF5A2 as a protein that upregulated PI3K/Akt and EMT pathways. MiR-1275 was expressed at a higher level by genistein treatment (Yang et al., 2022). Furthermore, it has been shown that miR-140-5p is involved in drug resistance in HCC cells. In brief, miR-140-5p improves DOX sensitivity through PIN1 depletion (Gao et al., 2021) and catalpol sensitivity through EMT suppression (Wu et al., 2021).

Cisplatin is a chemotherapeutic that inhibits transcription and replication, inducing apoptosis and necrosis in HCC cells (Ishikawa, 2009). It has been shown that miR-9 increases cisplatin sensitivity *in vitro* and *in vivo* by targeting EIF5A2 and EMT process. Besides that, EIF5A2 depletion decreases vimentin expression and increases E-cadherin in HCC cell lines (Bao et al., 2020). Another ncRNA involved in cisplatin sensitivity is miR-138 by its direct target, enhancer of zeste homolog 2 (EZH2). This miRNA upregulates EMT markers; therefore, the miR-138/EZH2/EMT axis could regulate cisplatin resistance (Zeng et al., 2021), also involved in radiosensitivity. Bai et al. (2022) show that miR-138 is downregulated in HCC tissue and its expression is indirectly correlated with EZH2 expression, which is a direct target of miR-138-5p. By RNA-seq, they observed that miR-138-5p upregulation inhibits HIF-1α and EMT (Bai et al., 2022). Moreover, Lu et al. (2022) reported that miR-138-5p is negatively regulated by circ-TLK1.

**FIGURE 2:**
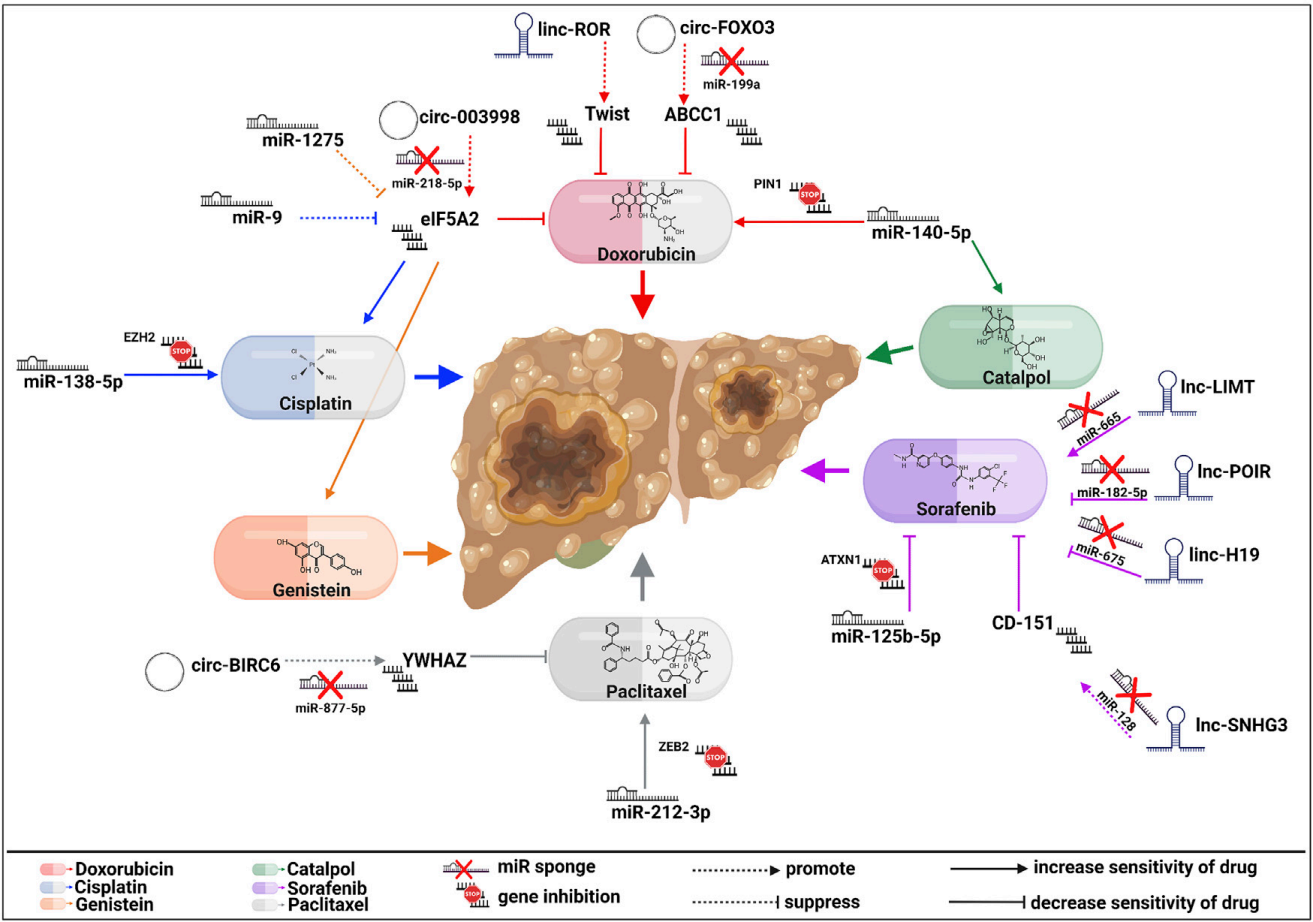
Molecular actions of EMT-related ncRNA axes in HCC drug resistance. Multiple regulatory components either increase or decrease sensitivity to sorafenib, paclitaxel, genistein, cisplatin, doxorubicin, or catalpol, affecting HCC progression. The signaling pathways of every drug are represented by different colors, as seen above (created with biorender.com accessed on July 2023).

Paclitaxel—a microtubule-stabilizing molecule, induces cell death (Weaver, 2014). As mentioned above, paclitaxel (PTX) is another drug whose resistance could be caused by different signaling pathways, including ncRNAs and EMT (Ashrafizadeh et al., 2021). Liu et al. (2020) pointed out circ-BIRC6 (circRNA baculoviral IAP repeat-containing 6) as an inhibitor of PTX sensitivity by sponging miR-8 77-5p to enhance tyrosine 3-monooxygenase/tryptophan 5-monooxygenase activation protein, zeta (YWHAZ) expression. Its role in drug resistance has been reported in ovarian cancer (Hong et al., 2018), bladder cancer (Yu et al., 2019), and gastric cancer (Zhao et al., 2021). Furthermore, miR-212-3p is decreased in PTX-resistant cells. This miRNA can bind to 3′UTR ZEB2, thus mediating chemoresistance in HCC cells. Transfection of miR-212-3p in resistant cells inhibited ZEB2 expression, reversing EMT (Yang et al., 2020). Figure 2 summarizes the ncRNAs axes involved in HCC drug resistance.

These investigations show the complex and dual role of ncRNAs in EMT. The exact mechanism by which every ncRNA is involved in the HCC will be difficult to decode because of its functions in many hepatocellular processes. One way to start is by classifying the miRNAs based on their direct or indirect impact on the EMT process. Undoubtedly, future studies are necessary to report new miRNAs associated with HCC-EMT and to map their function in this process, which can lead to the development of novel therapies.

Therefore, to translate ncRNAs in a therapeutic situation, tools must be developed to analyze these ncRNA axes functionally and to devise therapy strategies, so as to overcome off-target and toxicity consequences.

## 4 EMT-associated exosomal ncRNAs in HCC

Exosomes can be found in all human body fluids (blood, urine, saliva, ascites, cerebrospinal and synovial fluids) (Jiang et al., 2022). They are extracellular 30–100 nm vesicles (EVs) having a lipid bilayer; they are generated from the luminal membranes of multivesicular bodies (MVBs) and released into the extracellular matrix after MVBs fusion with the cell membrane (Kim et al., 2020). The primary physiological role of exosomes is to mediate cell-cell communication by transferring bioactive molecules, such as proteins or nucleic acids (Chen et al., 2021), thus being one of the most studied tools for the interchange of substances between tumor cells and the tumor microenvironment (Jiang et al., 2022).

In the last decade, more studies have highlighted the regulatory effects of different bioactive molecules delivered by exosomes, such as ncRNAs, in the EMT process in various types of cancers, including HCC. Interestingly, they can promote or suppress the EMT phenomena in HCC cells.

According to RNAseq investigation, exosomal miR-92a-3p expression level increases in two established high-metastatic HCC cell lines (97 hm and Huhm). Besides, treatment with high-metastatic HCC-derived exosomal miR-92a-3p facilitates the aggressiveness of HCC cells via PTEN inhibition and Akt/Snail signaling activation, promoting EMT (Yang et al., 2020). Similarly, high levels of miR-4800-3p were found in Huh7 cell-derived exosomes. Thus, Lin et al. (2022) demonstrated that exosomal miR-4800-3p heightened the progression of HCC by regulating the Hippo signaling pathway and targeting STK25 in both *in vitro* and *in vivo* experiments. Moreover, the treatment of low metastatic HCC cells with exosomal miR-4800-3p downregulates the expression of E-cadherin and ZO-1 and increases the expression of N-cadherin, activating the EMT process (Lin et al., 2022).

Interestingly, M2 macrophages can influence tumor development by secreting various cytokines and exosomes that can be loaded with specific miRNAs. For instance, miR-660-5p-loaded M2 exosomes augmented EMT and enhanced the tumorigenic ability in HCC cells through downregulating Kruppel-like factor 3 (KLF3) expression (Tian et al., 2021).

Human umbilical cord mesenchymal stem cells (hucMSCs) have low immunogenicity and high proliferation and differentiation potential. Additionally, the treatment of HCC cells with hucMSC-Exo upregulates miR-451a. This miRNA inhibits a disintegrin and metalloprotease 10 (ADAM10), thus reducing EMT and aggressive phenotypes of HCC (Xu et al., 2021).

Several studies showed that TGF-β treatment induces EMT (Miyazono, 2009; Lin et al., 2020; Kim et al., 2021) and treatment with exosomes derived from these cells increases proliferation and metastasis in HCC cells (Lin et al., 2020) through intercellular communication. Lin et al. (2020) reported that 119 miRNAs are upregulated, such as miR-125b-5p, 374a-5p, miR-24-3p, miR-200b-3p, and miR-21-5p, and 186 are downregulated in EMT-Hep3B-derived exosomes (EMT-Hep3B exo), as compared to Hep3B exo. Moreover, treatment with EMT-Hep3B exo with miR-374a-5p interference inhibits hepatocellular metastasis by upregulation of growth arrest and DNA damage 45-alpha (GADD45A), a cell growth suppressor (Lin et al., 2020). In contrast, Huh7 cell-derived exosomes loaded with miR-125b (Exo-125b) blocks EMT and suppresses metastatic potential via inhibiting TGF-β1/SMAD pathways (Kim et al., 2021). Similarly, miR-374c-5p was found to be downregulated in the EMT model and transferred by exosomes derived from bone marrow mesenchymal stem cells (BMSC) suppresses EMT via targeting LIM domain kinase 1 (LIMK1) and inhibiting Wnt/β-catenin and TGF-β1 axes in HCC cells (Ding et al., 2023).

Yao et al. (2022) identified that lncRNA THEMIS2-211 is upregulated in plasma-derived exosomes from HCC patients. Knockdown of THEMIS2-211 increases E-cadherin and decreases N-cadherin and vimentin in HCC cells. Mechanistically, they showed that THEMIS2-211 is an oncogene that promotes proliferation, migration, invasion, and EMT by sponging miR-940 and increasing SPOCK1 expression (Yao et al., 2022).

Circ-0004277 and lncRNA PRR34-AS1 transfer via exosomes to human hepatic cells increases the malignant phenotype (Zhu et al., 2020; Zhang et al., 2022). Thus, PRR34-AS1 enhanced Rab27a expression to increase the exosome secretion of VEGF and TGF-β in HCC cells and transmitted them into the human liver epithelial (THLE-3) cells (Zhang et al., 2022).

In summary, these studies prove that exosomes act as ncRNAs cargo for tumor cells and have distinct regulatory effects on the EMT process in HCC and various underlying processes. Although exosomes are promising therapy in cancer, improvement of their purification, and additional studies on the interaction and mechanisms with other types of cells remain the main problems to be solved in their uses.

## 5 Conclusion and future perspectives

The development of transcriptomics approaches in the last decade has highlighted the essential roles of ncRNAs in cancer (Slack and Chinnaiyan, 2019; Winkle et al., 2021). The formation of ncRNA axes starts to become an essential tool in various cellular mechanisms, and its role in the progression of HCC is decisive (Wong et al., 2018). Furthermore, it will be crucial to comprehend how ncRNA axes regulate migration, proliferation, and EMT in HCC cells, so as to generate cutting-edge therapeutic medications based on ncRNAs, to prevent and manage HCC.

Taking together these observations, we find that defining ncRNA pathways in direct and indirect mechanisms could map a precise road to a therapeutic target as close to a clinical necessity as possible. The EMT-related miRNAs’ direct mechanism of action could be a promissive path in developing new therapies against metastasis. However, more research is needed to understand how these miRNA axes work and to determine which transcripts are valuable targets. Undoubtedly, since a single miRNA could have several targets and can affect more therapeutic drugs, its use as a new therapy in cancer requires an in-depth study of the mechanisms involved.

## References

[B1] Ashrafizadeh M. Mirzaei S. Hashemi F. Zarrabi A. Zabolian A. Saleki H. (2021). New insight towards development of paclitaxel and docetaxel resistance in cancer cells: EMT as a novel molecular mechanism and therapeutic possibilities. Biomed. Pharmacother. 141, 111824. 10.1016/j.biopha.2021.111824 34175815

[B2] Ayesha M. Majid A. Zhao D. Greenaway F. T. Yan N. Liu Q. (2022). MiR-4521 plays a tumor repressive role in growth and metastasis of hepatocarcinoma cells by suppressing phosphorylation of FAK/AKT pathway via targeting FAM129A. J. Adv. Res. 36, 147–161. 10.1016/j.jare.2021.05.003 35127170 PMC8799875

[B3] Bai B. Liu Y. Fu X. M. Qin H. Y. Li G. K. Wang H. C. (2022). Dysregulation of EZH2/miR-138-5p Axis contributes to radiosensitivity in hepatocellular carcinoma cell by downregulating hypoxia-inducible factor 1 alpha (HIF-1*α*). Oxid. Med. Cell Longev. 2022, 7608712. 10.1155/2022/7608712 36071871 PMC9444475

[B4] Bakir B. Chiarella A. M. Pitarresi J. R. Rustgi A. K. (2020). EMT, MET, plasticity, and tumor metastasis. Trends Cell Biol. 30 (10), 764–776. 10.1016/j.tcb.2020.07.003 32800658 PMC7647095

[B5] Bao Y. Zhang Y. Lu Y. Guo H. Dong Z. Chen Q. (2020). Overexpression of microRNA-9 enhances cisplatin sensitivity in hepatocellular carcinoma by regulating EIF5A2-mediated epithelial-mesenchymal transition. Int. J. Biol. Sci. 16 (5), 827–837. 10.7150/ijbs.32460 32071552 PMC7019138

[B6] Bartel D. P. (2004). MicroRNAs: genomics, biogenesis, mechanism, and function. Cell 116 (2), 281–297. 10.1016/s0092-8674(04)00045-5 14744438

[B7] Bassett A. R. Azzam G. Wheatley L. Tibbit C. Rajakumar T. McGowan S. (2014). Understanding functional miRNA-target interactions *in vivo* by site-specific genome engineering. Nat. Commun. 5, 4640. 10.1038/ncomms5640 25135198 PMC4143950

[B8] Brockhausen J. Tay S. S. Grzelak C. A. Bertolino P. Bowen D. G. d'Avigdor W. M. (2015). miR-181a mediates TGF-beta-induced hepatocyte EMT and is dysregulated in cirrhosis and hepatocellular cancer. Liver Int. 35 (1), 240–253. 10.1111/liv.12517 24576072

[B9] Cai X. Hagedorn C. H. Cullen B. R. (2004). Human microRNAs are processed from capped, polyadenylated transcripts that can also function as mRNAs. RNA 10 (12), 1957–1966. 10.1261/rna.7135204 15525708 PMC1370684

[B10] Cao C. Li J. Li G. Hu G. Deng Z. Huang B. (2021). Long non-coding RNA TMEM220-AS1 suppressed hepatocellular carcinoma by regulating the miR-484/MAGI1 Axis as a competing endogenous RNA. Front. Cell Dev. Biol. 9, 681529. 10.3389/fcell.2021.681529 34422806 PMC8376477

[B11] Chen B. W. Zhou Y. Wei T. Wen L. Zhang Y. B. Shen S. C. (2021). lncRNA-POIR promotes epithelial-mesenchymal transition and suppresses sorafenib sensitivity simultaneously in hepatocellular carcinoma by sponging miR-182-5p. J. Cell Biochem. 122 (1), 130–142. 10.1002/jcb.29844 32951268

[B12] Chen F. He L. Qiu L. Zhou Y. Li Z. Chen G. (2021). Circular RNA CircEPB41L2 functions as tumor suppressor in hepatocellular carcinoma through sponging miR-590-5p. Cancer Manag. Res. 13, 2969–2981. 10.2147/CMAR.S291682 33833580 PMC8021265

[B13] Chen H. Wang L. Zeng X. Schwarz H. Nanda H. S. Peng X. (2021). Exosomes, a new star for targeted delivery. Front. Cell Dev. Biol. 9, 751079. 10.3389/fcell.2021.751079 34692704 PMC8531489

[B14] Chen J. Zhao Y. Zhang F. Li J. Boland J. A. Cheng N. C. (2022). Liver-specific deletion of miR-181ab1 reduces liver tumour progression via upregulation of CBX7. Cell Mol. Life Sci. 79 (8), 443. 10.1007/s00018-022-04452-6 35867177 PMC9307539

[B15] Chen L. L. (2016). The biogenesis and emerging roles of circular RNAs. Nat. Rev. Mol. Cell Biol. 17 (4), 205–211. 10.1038/nrm.2015.32 26908011

[B16] Chen P. P. Zhang Z. S. Wu J. C. Zheng J. F. Lin F. (2021). LncRNA SNHG12 promotes proliferation and epithelial mesenchymal transition in hepatocellular carcinoma through targeting HEG1 via miR-516a-5p. Cell Signal 84, 109992. 10.1016/j.cellsig.2021.109992 33774129

[B17] Chen T. Liu R. Niu Y. Mo H. Wang H. Lu Y. (2021). HIF-1α-activated long non-coding RNA KDM4A-AS1 promotes hepatocellular carcinoma progression via the miR-411-5p/KPNA2/AKT pathway. Cell Death Dis. 12 (12), 1152. 10.1038/s41419-021-04449-2 34903711 PMC8668937

[B18] Chen Y. Zhao J. Jiao Z. Wang W. Wang D. Yu X. (2018). SKA1 overexpression is associated with poor prognosis in hepatocellular carcinoma. BMC Cancer 18 (1), 1240. 10.1186/s12885-018-5119-6 30537941 PMC6288885

[B19] Couri T. Pillai A. (2019). Goals and targets for personalized therapy for HCC. Hepatol. Int. 13 (2), 125–137. 10.1007/s12072-018-9919-1 30600478

[B20] Cox J. Weinman S. (2016). Mechanisms of doxorubicin resistance in hepatocellular carcinoma. Hepat. Oncol. 3 (1), 57–59. 10.2217/hep.15.41 26998221 PMC4792121

[B21] Cui S. Chen Y. Guo Y. Wang X. Chen D. (2023). Hsa-miR-22-3p inhibits liver cancer cell EMT and cell migration/invasion by indirectly regulating SPRY2. PLoS One 18 (2), e0281536. 10.1371/journal.pone.0281536 36749775 PMC9904474

[B22] De Las Rivas J. Brozovic A. Izraely S. Casas-Pais A. Witz I. P. Figueroa A. (2021). Cancer drug resistance induced by EMT: Novel therapeutic strategies. Arch. Toxicol. 95 (7), 2279–2297. 10.1007/s00204-021-03063-7 34003341 PMC8241801

[B23] Ding B. Lou W. Fan W. Pan J. (2023). Exosomal miR-374c-5p derived from mesenchymal stem cells suppresses epithelial-mesenchymal transition of hepatocellular carcinoma via the LIMK1-Wnt/β-catenin axis. Environ. Toxicol. 38 (5), 1038–1052. 10.1002/tox.23746 36722453

[B24] Dudas J. Ladanyi A. Ingruber J. Steinbichler T. B. Riechelmann H. (2020). Epithelial to mesenchymal transition: A mechanism that fuels cancer radio/chemoresistance. Cells 9 (2), 428. 10.3390/cells9020428 32059478 PMC7072371

[B25] Esquela-Kerscher A. Slack F. J. (2006). Oncomirs - microRNAs with a role in cancer. Nat. Rev. Cancer 6 (4), 259–269. 10.1038/nrc1840 16557279

[B26] Feng K. L. Diao N. Zhou Z. W. Fang C. K. Wang J. N. Zhang Y. (2022). CircFGGY inhibits cell growth, invasion and epithelial-mesenchymal transition of hepatocellular carcinoma via regulating the miR-545-3p/smad7 Axis. Front. Cell Dev. Biol. 10, 850708. 10.3389/fcell.2022.850708 35592246 PMC9110866

[B27] Feng Y. Wei G. Zhang L. Zhou H. Wang W. Guo P. (2021). LncRNA DARS-AS1 aggravates the growth and metastasis of hepatocellular carcinoma via regulating the miR-3200-5p-Cytoskeleton associated protein 2 (CKAP2) axis. Bioengineered 12 (1), 8217–8232. 10.1080/21655979.2021.1982272 34596006 PMC8806480

[B28] Gao X. Jiang Y. Li Y. (2021). Inhibitory effect of miR-140-5p on doxorubicin resistance of hepatocellular carcinoma. Exp. Ther. Med. 21 (5), 507. 10.3892/etm.2021.9938 33791016 PMC8005744

[B29] Gong T. T. Sun F. Z. Chen Y. J. Liu J. F. Yan Y. Li D. (2020). The circular RNA circPTK2 inhibits EMT in hepatocellular carcinoma by acting as a ceRNA and sponging miR-92a to upregulate E-cadherin. Eur. Rev. Med. Pharmacol. Sci. 24 (18), 9333–9342. 10.26355/eurrev_202009_23015 33015774

[B30] Guan Q. Yuan B. Zhang X. Yan T. Li J. Xu W. (2021). Long non-coding RNA DUXAP8 promotes tumorigenesis by regulating IGF1R via miR-9-3p in hepatocellular carcinoma. Exp. Ther. Med. 22 (1), 755. 10.3892/etm.2021.10187 34035852 PMC8135127

[B31] Ha M. Kim V. N. (2014). Regulation of microRNA biogenesis. Nat. Rev. Mol. Cell Biol. 15 (8), 509–524. 10.1038/nrm3838 25027649

[B32] Hao Y. Baker D. Ten Dijke P. (2019). TGF-beta-Mediated epithelial-mesenchymal transition and cancer metastasis. Int. J. Mol. Sci. 20 (11), 2767. 10.3390/ijms20112767 31195692 PMC6600375

[B33] He Q. Guo P. Bo Z. Yu H. Yang J. Wang Y. (2022). Noncoding RNA-mediated molecular bases of chemotherapy resistance in hepatocellular carcinoma. Cancer Cell Int. 22 (1), 249. 10.1186/s12935-022-02643-6 35945536 PMC9361533

[B34] Hirao A. Sato Y. Tanaka H. Nishida K. Tomonari T. Hirata M. (2021). MiR-125b-5p is involved in sorafenib resistance through ataxin-1-mediated epithelial-mesenchymal transition in hepatocellular carcinoma. Cancers (Basel) 13 (19), 4917. 10.3390/cancers13194917 34638401 PMC8508441

[B35] Hong L. Chen W. Xing A. Wu D. Wang S. (2018). Inhibition of tyrosine 3-monooxygenase/tryptophan 5-monooxygenase activation protein zeta (YWHAZ) overcomes drug resistance and tumorigenicity in ovarian cancer. Cell Physiol. Biochem. 49 (1), 53–64. 10.1159/000492839 30134224

[B36] Huang J. Yang Y. Zhao F. Zhang Z. Deng J. Lu W. (2023). LncRNA SATB2-AS1 overexpression represses the development of hepatocellular carcinoma through regulating the miR-3678-3p/GRIM-19 axis. Cancer Cell Int. 23 (1), 82. 10.1186/s12935-023-02901-1 37118800 PMC10148439

[B37] Huang W. Huang F. Feng C. (2020). CircFoxo3 promotes adriamycin resistance through regulation of miR-199a-5p/ATP binding cassette subfamily C member 1 Axis in hepatocellular carcinoma. Onco Targets Ther. 13, 5113–5122. 10.2147/OTT.S243571 32606732 PMC7292492

[B38] Huang W. J. Tian X. P. Bi S. X. Zhang S. R. He T. S. Song L. Y. (2020). The beta-catenin/TCF-4-LINC01278-miR-1258-Smad2/3 axis promotes hepatocellular carcinoma metastasis. Oncogene 39 (23), 4538–4550. 10.1038/s41388-020-1307-3 32372060 PMC7269911

[B39] Huang X. Pan J. Wang G. Huang T. Li C. Wang Y. (2021). UNC5B-AS1 promotes the proliferation, migration and EMT of hepatocellular carcinoma cells via regulating miR-4306/KDM2A axis. Cell Cycle 20 (20), 2114–2124. 10.1080/15384101.2021.1962632 34612138 PMC8565830

[B40] Huang Y. Hong W. Wei X. (2022). The molecular mechanisms and therapeutic strategies of EMT in tumor progression and metastasis. J. Hematol. Oncol. 15 (1), 129. 10.1186/s13045-022-01347-8 36076302 PMC9461252

[B41] Ishikawa T. (2009). Future perspectives on the treatment of hepatocellular carcinoma with cisplatin. World J. Hepatol. 1 (1), 8–16. 10.4254/wjh.v1.i1.8 21160960 PMC2998955

[B42] Ji J. Yamashita T. Budhu A. Forgues M. Jia H. L. Li C. (2009). Identification of microRNA-181 by genome-wide screening as a critical player in EpCAM-positive hepatic cancer stem cells. Hepatology 50 (2), 472–480. 10.1002/hep.22989 19585654 PMC2721019

[B43] Jiang C. Zeng X. Shan R. Wen W. Li J. Tan J. (2020). The emerging picture of the roles of CircRNA-CDR1as in cancer. Front. Cell Dev. Biol. 8, 590478. 10.3389/fcell.2020.590478 33335899 PMC7736612

[B44] Jiang J. Li J. Zhou X. Zhao X. Huang B. Qin Y. (2022). Exosomes regulate the epithelial-mesenchymal transition in cancer. Front. Oncol. 12, 864980. 10.3389/fonc.2022.864980 35359397 PMC8964004

[B45] Jin J. Liu H. Jin M. Li W. Xu H. Wei F. (2020). Silencing of hsa_circ_0101145 reverses the epithelial-mesenchymal transition in hepatocellular carcinoma via regulation of the miR-548c-3p/LAMC2 axis. Aging (Albany NY) 12 (12), 11623–11635. 10.18632/aging.103324 32554866 PMC7343517

[B46] Ju L. Yao M. Lu R. Cao Y. Wang H. Yuan L. (2022). Circular RNA hsa_circ_0051040 promotes hepatocellular carcinoma progression by sponging miR-569 and regulating ITGAV expression. Cells 11 (22), 3571. 10.3390/cells11223571 36429000 PMC9688127

[B47] Khanbabaei H. Ebrahimi S. Garcia-Rodriguez J. L. Ghasemi Z. Pourghadamyari H. Mohammadi M. (2022). Non-coding RNAs and epithelial mesenchymal transition in cancer: Molecular mechanisms and clinical implications. J. Exp. Clin. Cancer Res. 41 (1), 278. 10.1186/s13046-022-02488-x 36114510 PMC9479306

[B48] Kim H. Lee S. Shin E. Seong K. M. Jin Y. W. Youn H. (2020). The emerging roles of exosomes as EMT regulators in cancer. Cells 9 (4), 861. 10.3390/cells9040861 32252322 PMC7226841

[B49] Kim H. S. Kim J. S. Park N. R. Nam H. Sung P. S. Bae S. H. (2021). Exosomal miR-125b exerts anti-metastatic properties and predicts early metastasis of hepatocellular carcinoma. Front. Oncol. 11, 637247. 10.3389/fonc.2021.637247 34386414 PMC8354570

[B50] Klein T. Bischoff R. (2011). Physiology and pathophysiology of matrix metalloproteases. Amino Acids 41 (2), 271–290. 10.1007/s00726-010-0689-x 20640864 PMC3102199

[B51] Kristensen L. S. Andersen M. S. Stagsted L. V. W. Ebbesen K. K. Hansen T. B. Kjems J. (2019). The biogenesis, biology and characterization of circular RNAs. Nat. Rev. Genet. 20 (11), 675–691. 10.1038/s41576-019-0158-7 31395983

[B52] Li D. Zhou T. Li Y. Xu Y. Cheng X. Chen J. (2022). LINC02362 attenuates hepatocellular carcinoma progression through the miR-516b-5p/SOSC2 axis. Aging (Albany NY) 14 (1), 368–388. 10.18632/aging.203813 34990401 PMC8791201

[B53] Li G. Du P. He J. Li Y. (2021). CircRNA circBACH1 (hsa_circ_0061395) serves as a miR-656-3p sponge to facilitate hepatocellular carcinoma progression through increasing SERBP1 expression. Biochem. Biophys. Res. Commun. 556, 1–8. 10.1016/j.bbrc.2021.03.136 33831787

[B54] Li M. Yue W. Li Q. Yu W. Li Y. Cao X. (2021). Circular RNA Circ_0000098 elevates ALX4 expression via adsorbing miR-1204 to inhibit the progression of hepatocellular carcinoma. Front. Oncol. 11, 696078. 10.3389/fonc.2021.696078 34900665 PMC8662564

[B55] Li N. L. Xiao G. Jin Y. Y. Deng Y. Y. Liu Y. J. Yin L. C. (2022). Long non-coding RNA LINC00992 promotes hepatocellular carcinoma cell proliferation, metastasis, and invasiveness by downregulating MicroRNA miR-361-5p expression to increase levels of the transcription factor twist1. Pathol. Res. Pract. 238, 154115. 10.1016/j.prp.2022.154115 36084427

[B56] Li W. Xue H. Li Y. Li P. Ma F. Liu M. (2021). HIPK3 circular RNA promotes metastases of HCC through sponging miR-338-3p to induce ZEB2 expression. Dig. Dis. Sci. 66 (10), 3439–3447. 10.1007/s10620-020-06688-3 33247421

[B57] Li X. He J. Ren X. Zhao H. Zhao H. (2020). Circ_0003998 enhances doxorubicin resistance in hepatocellular carcinoma by regulating miR-218-5p/EIF5A2 pathway. Diagn Pathol. 15 (1), 141. 10.1186/s13000-020-01056-1 33308276 PMC7733254

[B58] Li Z. Wu G. Li J. Wang Y. Ju X. Jiang W. (2020). lncRNA CRNDE promotes the proliferation and metastasis by acting as sponge miR-539-5p to regulate POU2F1 expression in HCC. BMC Cancer 20 (1), 282. 10.1186/s12885-020-06771-y 32252678 PMC7137470

[B59] Liao Y. Yang D. Luo H. Yang G. Wen X. Zhang K. (2021). CircSEC24A promotes tumor progression through sequestering miR-455-3p in hepatocellular carcinoma. Neoplasma 2021, 210305N285. 10.4149/neo_2021_210305N285 34459207

[B60] Lin H. Peng J. Zhu T. Xiong M. Zhang R. Lei L. (2022). Exosomal miR-4800-3p aggravates the progression of hepatocellular carcinoma via regulating the Hippo signaling pathway by targeting STK25. Front. Oncol. 12, 759864. 10.3389/fonc.2022.759864 35756606 PMC9214204

[B61] Lin Q. Zhou C. R. Bai M. J. Zhu D. Chen J. W. Wang H. F. (2020). Exosome-mediated miRNA delivery promotes liver cancer EMT and metastasis. Am. J. Transl. Res. 12 (3), 1080–1095. 32269736 PMC7137059

[B62] Liu C. Wang H. Tang L. Huang H. Xu M. Lin Y. (2021). LncRNA BACE1-AS enhances the invasive and metastatic capacity of hepatocellular carcinoma cells through mediating miR-377-3p/CELF1 axis. Life Sci. 275, 119288. 10.1016/j.lfs.2021.119288 33667514

[B63] Liu W. Gao X. Chen X. Zhao N. Sun Y. Zou Y. (2021). miR-139-5p loss-mediated WTAP activation contributes to hepatocellular carcinoma progression by promoting the epithelial to mesenchymal transition. Front. Oncol. 11, 611544. 10.3389/fonc.2021.611544 33937023 PMC8083052

[B64] Liu Y. Guo J. Shen K. Wang R. Chen C. Liao Z. (2020). Paclitaxel suppresses hepatocellular carcinoma tumorigenesis through regulating circ-BIRC6/miR-877-5p/YWHAZ Axis. Onco Targets Ther. 13, 9377–9388. 10.2147/OTT.S261700 33061425 PMC7519811

[B65] Liu Y. Song J. Zhang H. Liao Z. Liu F. Su C. (2022). EIF4A3-induced circTOLLIP promotes the progression of hepatocellular carcinoma via the miR-516a-5p/PBX3/EMT pathway. J. Exp. Clin. Cancer Res. 41 (1), 164. 10.1186/s13046-022-02378-2 35509064 PMC9069765

[B66] Long J. Liu L. Yang X. Zhou X. Lu X. Qin L. (2023). LncRNA NUTM2A-AS1 aggravates the progression of hepatocellular carcinoma by activating the miR-186-5p/KLF7-mediated Wnt/beta-catenin pathway. Hum. Cell 36 (1), 312–328. 10.1007/s13577-022-00802-5 36242728

[B67] Lu J. Wang X. Z. Zhang T. Q. Huang X. Y. Yao J. G. Wang C. (2017). Prognostic significance of XRCC4 expression in hepatocellular carcinoma. Oncotarget 8 (50), 87955–87970. 10.18632/oncotarget.21360 29152133 PMC5675685

[B68] Lu Y. Liu Y. Zhang K. Jiang L. (2022). Circular RNA TLK1 exerts oncogenic functions in hepatocellular carcinoma by acting as a ceRNA of miR-138-5p. J. Oncol. 2022, 2415836. 10.1155/2022/2415836 35359342 PMC8964207

[B69] Mao Y. Ding Z. Jiang M. Yuan B. Zhang Y. Zhang X. (2022). Circ_0091579 exerts an oncogenic role in hepatocellular carcinoma via mediating miR-136-5p/TRIM27. Biomed. J. 45 (6), 883–895. 10.1016/j.bj.2021.12.009 34974169 PMC9795369

[B70] Marisi G. Cucchetti A. Ulivi P. Canale M. Cabibbo G. Solaini L. (2018). Ten years of sorafenib in hepatocellular carcinoma: Are there any predictive and/or prognostic markers? World J. Gastroenterol. 24 (36), 4152–4163. 10.3748/wjg.v24.i36.4152 30271080 PMC6158485

[B71] Mattick J. S. Amaral P. P. Carninci P. Carpenter S. Chang H. Y. Chen L. L. (2023). Long non-coding RNAs: Definitions, functions, challenges and recommendations. Nat. Rev. Mol. Cell Biol. 24 (6), 430–447. 10.1038/s41580-022-00566-8 36596869 PMC10213152

[B72] Meng F. Liu J. Lu T. Zang L. Wang J. He Q. (2021). SNHG1 knockdown upregulates miR-376a and downregulates FOXK1/Snail axis to prevent tumor growth and metastasis in HCC. Mol. Ther. Oncolytics 21, 264–277. 10.1016/j.omto.2021.02.002 34095464 PMC8143978

[B73] Miyazono K. (2009). Transforming growth factor-beta signaling in epithelial-mesenchymal transition and progression of cancer. Proc. Jpn. Acad. Ser. B Phys. Biol. Sci. 85 (8), 314–323. 10.2183/pjab.85.314 PMC3621568 19838011

[B74] Mjelle R. Dima S. O. Bacalbasa N. Chawla K. Sorop A. Cucu D. (2019). Comprehensive transcriptomic analyses of tissue, serum, and serum exosomes from hepatocellular carcinoma patients. BMC Cancer 19 (1), 1007. 10.1186/s12885-019-6249-1 31660891 PMC6816220

[B75] Nadhan R. Isidoro C. Song Y. S. Dhanasekaran D. N. (2022). Signaling by LncRNAs: Structure, cellular homeostasis, and disease pathology. Cells 11 (16), 2517. 10.3390/cells11162517 36010595 PMC9406440

[B76] Nicoloso M. S. Spizzo R. Shimizu M. Rossi S. Calin G. A. (2009). MicroRNAs--the micro steering wheel of tumour metastases. Nat. Rev. Cancer 9 (4), 293–302. 10.1038/nrc2619 19262572

[B77] Niu M. Yi M. Li N. Wu K. Wu K. (2021). Advances of targeted therapy for hepatocellular carcinoma. Front. Oncol. 11, 719896. 10.3389/fonc.2021.719896 34381735 PMC8350567

[B78] O'Brien J. Hayder H. Zayed Y. Peng C. (2018). Overview of MicroRNA biogenesis, mechanisms of actions, and circulation. Front. Endocrinol. (Lausanne) 9, 402. 10.3389/fendo.2018.00402 30123182 PMC6085463

[B79] Park N. R. Cha J. H. Sung P. S. Jang J. W. Choi J. Y. Yoon S. K. (2022). MiR-23b-3p suppresses epithelial-mesenchymal transition, migration, and invasion of hepatocellular carcinoma cells by targeting c-MET. Heliyon 8 (10), e11135. 10.1016/j.heliyon.2022.e11135 36281372 PMC9586913

[B80] Peng J. Wu H. J. Zhang H. F. Fang S. Q. Zeng R. (2021). miR-143-3p inhibits proliferation and invasion of hepatocellular carcinoma cells by regulating its target gene FGF1. Clin. Transl. Oncol. 23 (3), 468–480. 10.1007/s12094-020-02440-5 32617870

[B81] Peng S. Chen Y. Li T. Mao J. Yang P. Zou B. (2022). Hsa-microRNA-370-3p targeting Snail and Twist1 suppresses IL-8/STAT3-driven hepatocellular carcinoma metastasis. Cancer Sci. 113 (12), 4120–4134. 10.1111/cas.15571 36083239 PMC9746033

[B82] Pratama M. Y. Pascut D. Massi M. N. Tiribelli C. (2019). The role of microRNA in the resistance to treatment of hepatocellular carcinoma. Ann. Transl. Med. 7 (20), 577. 10.21037/atm.2019.09.142 31807558 PMC6861820

[B83] Qin M. Meng Y. Luo C. He S. Qin F. Yin Y. (2021). lncRNA PRR34-AS1 promotes HCC development via modulating Wnt/β-catenin pathway by absorbing miR-296-5p and upregulating E2F2 and SOX12. Mol. Ther. Nucleic Acids 25, 37–52. 10.1016/j.omtn.2021.04.016 34168917 PMC8190132

[B84] Rinn J. L. Chang H. Y. (2012). Genome regulation by long noncoding RNAs. Annu. Rev. Biochem. 81, 145–166. 10.1146/annurev-biochem-051410-092902 22663078 PMC3858397

[B85] Roos W. P. Kaina B. (2013). DNA damage-induced cell death: from specific DNA lesions to the DNA damage response and apoptosis. Cancer Lett. 332 (2), 237–248. 10.1016/j.canlet.2012.01.007 22261329

[B86] Sarkar F. H. Li Y. (2002). Mechanisms of cancer chemoprevention by soy isoflavone genistein. Cancer Metastasis Rev. 21 (3-4), 265–280. 10.1023/a:1021210910821 12549765

[B87] Schattenberg J. M. Schuchmann M. Galle P. R. (2011). Cell death and hepatocarcinogenesis: Dysregulation of apoptosis signaling pathways. J. Gastroenterol. Hepatol. 26 (1), 213–219. 10.1111/j.1440-1746.2010.06582.x 21199533

[B88] Scheau C. Badarau I. A. Costache R. Caruntu C. Mihai G. L. Didilescu A. C. (2019). The role of matrix metalloproteinases in the epithelial-mesenchymal transition of hepatocellular carcinoma. Anal. Cell Pathol. (Amst) 2019, 9423907. 10.1155/2019/9423907 31886121 PMC6899323

[B89] Shen D. Zhao H. Y. Gu A. D. Wu Y. W. Weng Y. H. Li S. J. (2021). miRNA-10a-5p inhibits cell metastasis in hepatocellular carcinoma via targeting SKA1. Kaohsiung J. Med. Sci. 37 (9), 784–794. 10.1002/kjm2.12392 34002462 PMC11896280

[B90] Shen D. Zhao H. Zeng P. Ge M. Shrestha S. Zhao W. (2022). Circular RNA circ_0001459 accelerates hepatocellular carcinoma progression via the miR-6165/IGF1R axis. Ann. N. Y. Acad. Sci. 1512 (1), 46–60. 10.1111/nyas.14753 35199365 PMC9306989

[B91] Sidhu K. Kapoor N. R. Pandey V. Kumar V. (2015). The "macro" world of microRNAs in hepatocellular carcinoma. Front. Oncol. 5, 68. 10.3389/fonc.2015.00068 25859429 PMC4373247

[B92] Skovierova H. Okajcekova T. Strnadel J. Vidomanova E. Halasova E. (2018). Molecular regulation of epithelial-to-mesenchymal transition in tumorigenesis (Review). Int. J. Mol. Med. 41 (3), 1187–1200. 10.3892/ijmm.2017.3320 29286071 PMC5819928

[B93] Slack F. J. Chinnaiyan A. M. (2019). The role of non-coding RNAs in oncology. Cell 179 (5), 1033–1055. 10.1016/j.cell.2019.10.017 31730848 PMC7347159

[B94] Song G. Q. He T. L. Ji K. J. Duan Y. M. Zhang J. W. Hu G. Q. (2022). SKA1/2/3 is a biomarker of poor prognosis in human hepatocellular carcinoma. Front. Oncol. 12, 1038925. 10.3389/fonc.2022.1038925 36439516 PMC9684634

[B95] Song L. N. Qiao G. L. Yu J. Yang C. M. Chen Y. Deng Z. F. (2020). Hsa_circ_0003998 promotes epithelial to mesenchymal transition of hepatocellular carcinoma by sponging miR-143-3p and PCBP1. J. Exp. Clin. Cancer Res. 39 (1), 114. 10.1186/s13046-020-01576-0 32552766 PMC7302140

[B96] Song S. Qiu X. (2021). LncRNA miR503HG inhibits epithelial-mesenchymal transition and angiogenesis in hepatocellular carcinoma by enhancing PDCD4 via regulation of miR-15b. Dig. Liver Dis. 53 (1), 107–116. 10.1016/j.dld.2020.09.008 33046427

[B97] Sorop A. Iacob R. Iacob S. Constantinescu D. Chitoiu L. Fertig T. E. (2020). Plasma small extracellular vesicles derived miR-21-5p and miR-92a-3p as potential biomarkers for hepatocellular carcinoma screening. Front. Genet. 11, 712. 10.3389/fgene.2020.00712 32793278 PMC7391066

[B98] Statello L. Guo C. J. Chen L. L. Huarte M. (2021). Gene regulation by long non-coding RNAs and its biological functions. Nat. Rev. Mol. Cell Biol. 22 (2), 96–118. 10.1038/s41580-020-00315-9 33353982 PMC7754182

[B99] Sun J. Zheng X. Wang B. Cai Y. Zheng L. Hu L. (2022). LncRNA LIMT (LINC01089) contributes to sorafenib chemoresistance via regulation of miR-665 and epithelial to mesenchymal transition in hepatocellular carcinoma cells. Acta Biochim. Biophys. Sin. (Shanghai) 54 (2), 261–270. 10.3724/abbs.2021019 35130616 PMC9909357

[B100] Sun W. Yang Y. Xu C. Guo J. (2017). Regulatory mechanisms of long noncoding RNAs on gene expression in cancers. Cancer Genet. 216-217, 105–110. 10.1016/j.cancergen.2017.06.003 29025584

[B101] Sung H. Ferlay J. Siegel R. L. Laversanne M. Soerjomataram I. Jemal A. (2021). Global cancer statistics 2020: GLOBOCAN estimates of incidence and mortality worldwide for 36 cancers in 185 countries. CA Cancer J. Clin. 71 (3), 209–249. 10.3322/caac.21660 33538338

[B102] Tang G. Zhao H. Xie Z. Wei S. Chen G. (2022). Long non-coding RNA HAGLROS facilitates tumorigenesis and progression in hepatocellular carcinoma by sponging miR-26b-5p to up-regulate karyopherin α2 (KPNA2) and inactivate p53 signaling. Bioengineered 13 (3), 7829–7846. 10.1080/21655979.2022.2049472 35291921 PMC9208501

[B103] Tang W. Chen Z. Zhang W. Cheng Y. Zhang B. Wu F. (2020). The mechanisms of sorafenib resistance in hepatocellular carcinoma: theoretical basis and therapeutic aspects. Signal Transduct. Target Ther. 5 (1), 87. 10.1038/s41392-020-0187-x 32532960 PMC7292831

[B104] Tewey K. M. Rowe T. C. Yang L. Halligan B. D. Liu L. F. (1984). Adriamycin-induced DNA damage mediated by mammalian DNA topoisomerase II. Science 226 (4673), 466–468. 10.1126/science.6093249 6093249

[B105] Tian B. Zhou L. Wang J. Yang P. (2021). miR-660-5p-loaded M2 macrophages-derived exosomes augment hepatocellular carcinoma development through regulating KLF3. Int. Immunopharmacol. 101, 108157. 10.1016/j.intimp.2021.108157 34673296

[B106] Toden S. Zumwalt T. J. Goel A. (2021). Non-coding RNAs and potential therapeutic targeting in cancer. Biochim. Biophys. Acta Rev. Cancer 1875 (1), 188491. 10.1016/j.bbcan.2020.188491 33316377 PMC7856203

[B107] Toki N. Takahashi H. Zucchelli S. Gustincich S. Carninci P. (2020). Synthetic *in vitro* transcribed lncRNAs (SINEUPs) with chemical modifications enhance target mRNA translation. FEBS Lett. 594 (24), 4357–4369. 10.1002/1873-3468.13928 33012004

[B108] Valdes F. Alvarez A. M. Locascio A. Vega S. Herrera B. Fernandez M. (2002). The epithelial mesenchymal transition confers resistance to the apoptotic effects of transforming growth factor Beta in fetal rat hepatocytes. Mol. Cancer Res. 1 (1), 68–78. 12496370

[B109] Vancza L. Karaszi K. Peterfia B. Turiak L. Dezso K. Sebestyen A. (2022). SPOCK1 promotes the development of hepatocellular carcinoma. Front. Oncol. 12, 819883. 10.3389/fonc.2022.819883 35186754 PMC8853618

[B110] Volinia S. Calin G. A. Liu C. G. Ambs S. Cimmino A. Petrocca F. (2006). A microRNA expression signature of human solid tumors defines cancer gene targets. Proc. Natl. Acad. Sci. U. S. A. 103 (7), 2257–2261. 10.1073/pnas.0510565103 16461460 PMC1413718

[B111] Wang B. Hsu S. H. Majumder S. Kutay H. Huang W. Jacob S. T. (2010). TGFbeta-mediated upregulation of hepatic miR-181b promotes hepatocarcinogenesis by targeting TIMP3. Oncogene 29 (12), 1787–1797. 10.1038/onc.2009.468 20023698 PMC2845743

[B112] Wang J. Zhu Y. Ai X. Wan H. Jia W. Chu J. (2023). Long noncoding RNA 02027 inhibits proliferation, migration and invasion of hepatocellular carcinoma via miR-625-3p/PDLIM5 pathway. J. Gene Med. 25 (6), e3485. 10.1002/jgm.3485 36811210

[B113] Wang L. Sun L. Liu R. Mo H. Niu Y. Chen T. (2021). Long non-coding RNA MAPKAPK5-AS1/PLAGL2/HIF-1α signaling loop promotes hepatocellular carcinoma progression. J. Exp. Clin. Cancer Res. 40 (1), 72. 10.1186/s13046-021-01868-z 33596983 PMC7891009

[B114] Weaver B. A. (2014). How Taxol/paclitaxel kills cancer cells. Mol. Biol. Cell 25 (18), 2677–2681. 10.1091/mbc.E14-04-0916 25213191 PMC4161504

[B115] Winkle M. El-Daly S. M. Fabbri M. Calin G. A. (2021). Noncoding RNA therapeutics - challenges and potential solutions. Nat. Rev. Drug Discov. 20 (8), 629–651. 10.1038/s41573-021-00219-z 34145432 PMC8212082

[B116] Wong C. M. Tsang F. H. Ng I. O. (2018). Non-coding RNAs in hepatocellular carcinoma: Molecular functions and pathological implications. Nat. Rev. Gastroenterol. Hepatol. 15 (3), 137–151. 10.1038/nrgastro.2017.169 29317776

[B117] Wu L. Li H. Chen S. Wu X. Chen X. Wang F. (2021). Catalpol inhibits the proliferation, migration and metastasis of HCC cells by regulating miR-140-5p expression. Mol. Med. Rep. 23 (1), 29. 10.3892/mmr.2020.11667 33179108 PMC7673346

[B118] Wu M. Sun T. Xing L. (2020). Circ_0004913 inhibits cell growth, metastasis, and glycolysis by absorbing miR-184 to regulate HAMP in hepatocellular carcinoma. Cancer Biother Radiopharm. 2020. 10.1089/cbr.2020.3779 33021399

[B119] Xu G. Zhang P. Liang H. Xu Y. Shen J. Wang W. (2021). Circular RNA hsa_circ_0003288 induces EMT and invasion by regulating hsa_circ_0003288/miR-145/PD-L1 axis in hepatocellular carcinoma. Cancer Cell Int. 21 (1), 212. 10.1186/s12935-021-01902-2 33858418 PMC8048300

[B120] Xu Y. Lai Y. Cao L. Li Y. Chen G. Chen L. (2021). Human umbilical cord mesenchymal stem cells-derived exosomal microRNA-451a represses epithelial-mesenchymal transition of hepatocellular carcinoma cells by inhibiting ADAM10. RNA Biol. 18 (10), 1408–1423. 10.1080/15476286.2020.1851540 33206588 PMC8489916

[B121] Xu Y. Liu Y. Li Z. Li H. Li X. Yan L. (2020). Long non-coding RNA H19 is involved in sorafenib resistance in hepatocellular carcinoma by upregulating miR-675. Oncol. Rep. 44 (1), 165–173. 10.3892/or.2020.7608 32627034 PMC7251775

[B122] Xuan W. Zhou C. You G. (2020). LncRNA LINC00668 promotes cell proliferation, migration, invasion ability and EMT process in hepatocellular carcinoma by targeting miR-532-5p/YY1 axis. Biosci. Rep. 40 (5). 10.1042/BSR20192697 PMC7214398 32249890

[B123] Yan L. Xu F. Dai C. L. (2018). Relationship between epithelial-to-mesenchymal transition and the inflammatory microenvironment of hepatocellular carcinoma. J. Exp. Clin. Cancer Res. 37 (1), 203. 10.1186/s13046-018-0887-z 30157906 PMC6114477

[B124] Yang B. Feng X. Liu H. Tong R. Wu J. Li C. (2020). High-metastatic cancer cells derived exosomal miR92a-3p promotes epithelial-mesenchymal transition and metastasis of low-metastatic cancer cells by regulating PTEN/Akt pathway in hepatocellular carcinoma. Oncogene 39 (42), 6529–6543. 10.1038/s41388-020-01450-5 32917956 PMC7561497

[B125] Yang J. Antin P. Berx G. Blanpain C. Brabletz T. Bronner M. (2020). Guidelines and definitions for research on epithelial-mesenchymal transition. Nat. Rev. Mol. Cell Biol. 21 (6), 341–352. 10.1038/s41580-020-0237-9 32300252 PMC7250738

[B126] Yang J. Cui R. Liu Y. (2020). MicroRNA-212-3p inhibits paclitaxel resistance through regulating epithelial-mesenchymal transition, migration and invasion by targeting ZEB2 in human hepatocellular carcinoma. Oncol. Lett. 20 (4), 23. 10.3892/ol.2020.11884 32774496 PMC7406882

[B127] Yang L. Deng W. L. Zhao B. G. Xu Y. Wang X. W. Fang Y. (2022). FOXO3-induced lncRNA LOC554202 contributes to hepatocellular carcinoma progression via the miR-485-5p/BSG axis. Cancer Gene Ther. 29 (3-4), 326–340. 10.1038/s41417-021-00312-w 33654226 PMC8940625

[B128] Yang X. Jiang W. Kong X. Zhou X. Zhu D. Kong L. (2022). Genistein restricts the epithelial mesenchymal transformation (EMT) and stemness of hepatocellular carcinoma via upregulating miR-1275 to inhibit the EIF5A2/PI3K/Akt pathway. Biol. (Basel) 11 (10), 1383. 10.3390/biology11101383 PMC9598820 36290289

[B129] Yao J. Hua X. Shi J. Hu X. Lui K. He K. (2022). LncRNA THEMIS2-211, a tumor-originated circulating exosomal biomarker, promotes the growth and metastasis of hepatocellular carcinoma by functioning as a competing endogenous RNA. FASEB J. 36 (4), e22238. 10.1096/fj.202101564R 35224785

[B130] Ye Z. He Q. Wang Q. Lin Y. Cen K. Chen X. (2021). LINC00922 promotes the proliferation, migration, invasion and EMT process of liver cancer cells by regulating miR-424-5p/ARK5. Mol. Cell Biochem. 476 (10), 3757–3769. 10.1007/s11010-021-04196-0 34097192

[B131] Yin D. Hu Z. Q. Luo C. B. Wang X. Y. Xin H. Y. Sun R. Q. (2021). LINC01133 promotes hepatocellular carcinoma progression by sponging miR-199a-5p and activating annexin A2. Clin. Transl. Med. 11 (5), e409. 10.1002/ctm2.409 34047479 PMC8101537

[B132] Yin L. C. Xiao G. Zhou R. Huang X. P. Li N. L. Tan C. L. (2020). MicroRNA-361-5p inhibits tumorigenesis and the EMT of HCC by targeting Twist1. Biomed. Res. Int. 2020, 8891876. 10.1155/2020/8891876 33381597 PMC7762665

[B133] Yu C. C. Li C. F. Chen I. H. Lai M. T. Lin Z. J. Korla P. K. (2019). YWHAZ amplification/overexpression defines aggressive bladder cancer and contributes to chemo-/radio-resistance by suppressing caspase-mediated apoptosis. J. Pathol. 248 (4), 476–487. 10.1002/path.5274 30945298 PMC6767422

[B134] Yuan S. Si W. Zhuang K. Li Y. Zhang Y. Liu J. (2021). LncRNA UCID promotes hepatocellular carcinoma metastasis via stabilization of snail. Onco Targets Ther. 14, 725–736. 10.2147/OTT.S277951 33536764 PMC7850577

[B135] Zeng T. Luo L. Huang Y. Ye X. Lin J. (2021). Upregulation of miR-138 increases sensitivity to cisplatin in hepatocellular carcinoma by regulating EZH2. Biomed. Res. Int. 2021, 6665918. 10.1155/2021/6665918 33748276 PMC7960019

[B136] Zeng Z. Dong J. Li Y. Dong Z. Liu Z. Huang J. (2020). The expression level and diagnostic value of microRNA-22 in HCC patients. Artif. Cells Nanomed Biotechnol. 48 (1), 683–686. 10.1080/21691401.2019.1703723 32088997

[B137] Zhang B. Li F. Zhu Z. Ding A. Luo J. (2020). CircRNA CDR1as/miR-1287/raf1 Axis modulates hepatocellular carcinoma progression through MEK/ERK pathway. Cancer Manag. Res. 12, 8951–8964. 10.2147/CMAR.S252679 33061591 PMC7522432

[B138] Zhang B. Zhou J. (2022). CircSEC24A (hsa_circ_0003528) interference suppresses epithelial-mesenchymal transition of hepatocellular carcinoma cells via miR-421/MMP3 axis. Bioengineered 13 (4), 9049–9062. 10.1080/21655979.2022.2057761 35400271 PMC9161912

[B139] Zhang C. Guo F. Xu G. Ma J. Shao F. (2015). STAT3 cooperates with Twist to mediate epithelial-mesenchymal transition in human hepatocellular carcinoma cells. Oncol. Rep. 33 (4), 1872–1882. 10.3892/or.2015.3783 25653024

[B140] Zhang H. Liu S. Chen L. Sheng Y. Luo W. Zhao G. (2021). MicroRNA miR-509-3p inhibit metastasis and epithelial-mesenchymal transition in hepatocellular carcinoma. Bioengineered 12 (1), 2263–2273. 10.1080/21655979.2021.1932210 34115554 PMC8806452

[B141] Zhang L. Zhang Y. Shen D. Chen Y. Feng J. Wang X. (2022). RNA binding motif protein 3 promotes cell metastasis and epithelial-mesenchymal transition through STAT3 signaling pathway in hepatocellular carcinoma. J. Hepatocell. Carcinoma 9, 405–422. 10.2147/JHC.S351886 35592242 PMC9112182

[B142] Zhang N. Wang F. Zhu L. Chang R. Mok S. R. S. Peixoto R. D. (2023). Molecular mechanism of the miR-7/BCL2L1/P53 signaling axis regulating the progression of hepatocellular carcinoma. Ann. Transl. Med. 11 (1), 12. 10.21037/atm-22-5929 36760243 PMC9906214

[B143] Zhang P. F. Wang F. Wu J. Wu Y. Huang W. Liu D. (2019). LncRNA SNHG3 induces EMT and sorafenib resistance by modulating the miR-128/CD151 pathway in hepatocellular carcinoma. J. Cell Physiol. 234 (3), 2788–2794. 10.1002/jcp.27095 30132868

[B144] Zhang Y. Wang D. Zhu T. Yu J. Wu X. Lin W. (2021). CircPUM1 promotes hepatocellular carcinoma progression through the miR-1208/MAP3K2 axis. J. Cell Mol. Med. 25 (1), 600–612. 10.1111/jcmm.15998 33320435 PMC7810943

[B145] Zhang Y. Wang Y. (2021). Circular RNAs in hepatocellular carcinoma: Emerging functions to clinical significances. Front. Oncol. 11, 667428. 10.3389/fonc.2021.667428 34055634 PMC8160296

[B146] Zhang Y. Wu W. Sun Q. Ye L. Zhou D. Wang W. (2021). linc-ROR facilitates hepatocellular carcinoma resistance to doxorubicin by regulating TWIST1-mediated epithelial-mesenchymal transition. Mol. Med. Rep. 23 (5), 340. 10.3892/mmr.2021.11979 33760121 PMC7974311

[B147] Zhang Z. Zhou Y. Jia Y. Wang C. Zhang M. Xu Z. (2022). PRR34-AS1 promotes exosome secretion of VEGF and TGF-beta via recruiting DDX3X to stabilize Rab27a mRNA in hepatocellular carcinoma. J. Transl. Med. 20 (1), 491. 10.1186/s12967-022-03628-9 36303180 PMC9615160

[B148] Zhao J. Fu X. Chen H. Min L. Sun J. Yin J. (2021). G3BP1 interacts with YWHAZ to regulate chemoresistance and predict adjuvant chemotherapy benefit in gastric cancer. Br. J. Cancer 124 (2), 425–436. 10.1038/s41416-020-01067-1 32989225 PMC7852868

[B149] Zhou Y. Li K. Dai T. Wang H. Hua Z. Bian W. (2021). Long non-coding RNA HCP5 functions as a sponge of miR-29b-3p and promotes cell growth and metastasis in hepatocellular carcinoma through upregulating DNMT3A. Aging (Albany NY) 13 (12), 16267–16286. 10.18632/aging.203155 34148029 PMC8266334

[B150] Zhu C. Su Y. Liu L. Wang S. Liu Y. Wu J. (2020). Circular RNA hsa_circ_0004277 stimulates malignant phenotype of hepatocellular carcinoma and epithelial-mesenchymal transition of peripheral cells. Front. Cell Dev. Biol. 8, 585565. 10.3389/fcell.2020.585565 33511111 PMC7835424

[B151] Zhu X. Jiang S. Wu Z. Liu T. Zhang W. Wu L. (2021). Long non-coding RNA TTN antisense RNA 1 facilitates hepatocellular carcinoma progression via regulating miR-139-5p/SPOCK1 axis. Bioengineered 12 (1), 578–588. 10.1080/21655979.2021.1882133 33517826 PMC8291788

